# Location Prediction Based on Transition Probability Matrices Constructing from Sequential Rules for Spatial-Temporal K-Anonymity Dataset

**DOI:** 10.1371/journal.pone.0160629

**Published:** 2016-08-10

**Authors:** Haitao Zhang, Zewei Chen, Zhao Liu, Yunhong Zhu, Chenxue Wu

**Affiliations:** 1 School of Geographic and Biological Information, Nanjing University of Posts and Telecommunications, Nanjing, China; 2 School of Telecommunications and Information Engineering, Nanjing University of Posts and Telecommunications, Nanjing, China; Beihang University, CHINA

## Abstract

Spatial-temporal k-anonymity has become a mainstream approach among techniques for protection of users’ privacy in location-based services (LBS) applications, and has been applied to several variants such as LBS snapshot queries and continuous queries. Analyzing large-scale spatial-temporal anonymity sets may benefit several LBS applications. In this paper, we propose two location prediction methods based on transition probability matrices constructing from sequential rules for spatial-temporal k-anonymity dataset. First, we define single-step sequential rules mined from sequential spatial-temporal k-anonymity datasets generated from continuous LBS queries for multiple users. We then construct transition probability matrices from mined single-step sequential rules, and normalize the transition probabilities in the transition matrices. Next, we regard a mobility model for an LBS requester as a stationary stochastic process and compute the n-step transition probability matrices by raising the normalized transition probability matrices to the power n. Furthermore, we propose two location prediction methods: rough prediction and accurate prediction. The former achieves the probabilities of arriving at target locations along simple paths those include only current locations, target locations and transition steps. By iteratively combining the probabilities for simple paths with n steps and the probabilities for detailed paths with n-1 steps, the latter method calculates transition probabilities for detailed paths with n steps from current locations to target locations. Finally, we conduct extensive experiments, and correctness and flexibility of our proposed algorithm have been verified.

## Introduction

With the rapid development in mobile communication and the popularity of positioning devices (e.g. Global Position System, GPS), LBS are widely used because of simplification in computing [1]. However, the deployment of LBS would bring privacy problems (e.g., employers snoop whereabouts of the staff, stalkers attack user trajectories to find out their religion, sex orientation, etc.) if used illegally, which has raised great attention from academia to business circle [2][3].

Early research on privacy protection for LBS users put emphasis on establishment of laws and treaties. While this research lacks flexibility, and has lagged behind attack technologies, some new technologies have been put forward. For instance, the use of hierarchical clustering [4], dummies [5][6], spatial transformation based on the Hilbert curve [7], private information retrieval (PIR) protocols [8] and spatial-temporal k-anonymity [9]. Spatial-temporal k-anonymity has become a mainstream privacy protection method for LBS users due to its simplification and various applications.

Furthermore, the basic principle of cloaking a requestor’s identification as well as accurate time and position information has inspired several variants on the original method [10].

As spatial and temporal properties are most important elements of spatial-temporal k-anonymity datasets (hereafter referred to as anonymity datasets), anonymity datasets can be formatted into a number of sequences of generalized regions. Analyzing large-scale anonymity datasets recorded and stored by LBS providers (such as Google Maps, Foursquare, Baidu Maps, etc.), can achieve a set of sequential rules reflecting LBS issuers’ movement behaviors. Furthermore, the sequential rules can be utilized to predict locations of future users, and provide assist decision support functions for LBS applications, such as intelligent navigation systems, personalized service systems, and so on [11][12]. Unfortunately, location prediction simply based on sequential rules does not perform well, as the prediction can only be single step, that is, the prediction only includes one source and one destination. A more practical location prediction method (such as multistep, etc.), is urgently needed in applications. To our knowledge, there is little literature that focuses on this subject by far.

In this paper, based on sequential rules mined from large-scale anonymity datasets, we propose two location prediction methods. Simultaneously, privacy attack problems that may result from our proposed location prediction methods are also analyzed.

The rest of this paper is organized as follows. Preliminary work is described in Section 2. Two location prediction methods based on preprocessing sequential rules from anonymity datasets are presented in Section 3. Comprehensive experiments are conducted in Section 4, and the results are analyzed. Section 5 concludes the paper and discusses further work.

### Preliminaries

In this section, the basic concepts of LBS queries and the primitives of LBS privacy are introduced. Examples of anonymity datasets adopted by a typical method of spatial-temporal k-anonymity are also presented.

### LBS query

A location service can be defined as a service that integrates the location of an LBS user with other information to provide added value to the user. Applications are designed by adopting two modes: push and pull [13]. Furthermore, there are two types of pull services, namely snapshot queries such as “recommend 10 nearby restaurants based on my profile”, and continuous queries such as “continually tell me the shopping mall nearest my location”. For a snapshot query, an LBS user only needs to report their current location to the service provider once to obtain the desired information. On the other hand, for a continuous query, an LBS user has to continually report their location to the service provider in a periodic or on-demand manner to obtain the desired results[14]. Additionally, in a continuous query, a consistent user identity (or pseudo-identifier) is used until the query expires, that is, LBS providers can link requests issued by the same (anonymous) user at different times in chronological order to obtain a sequence of requests.

### Primitives of LBS privacy

Privacy is an essential requirement for providing LBS, and can be grouped into two categories: identity and sensitive information [2]. Identity of each individual is unique which distinguishes an individual from a group of individuals (i.e., a security identifier or SID). Sensitive information consists of location and request content. Location privacy is the tracks of individuality or a group of people, which includes coordinates, landmarks, etc. Semantic location privacy is an instance of privacy regarding sensitive semantic information, for example, hospitals, religious buildings, and so on. Request content privacy involves sensitive attribute information, such as disease, salary, religion, and so on. It is worth noting that identity privacy can be associated with sensitive information privacy to cause more severe privacy invasion.

### Spatial-temporal k-anonymity

Spatial-temporal k-anonymity is a branch of the k-anonymity method, which is an obfuscation technique. Based on spatial-temporal k-anonymity, a query request submitted to LBS providers does not only contributed by the identity and location of LBS users, but also at least k pseudonyms of the users, including the requestor and others nearby, and a cloaking region enclosing the locations of the k (or more) LBS users. Thus, given a query request, an anonymity dataset is generated, consisting of at least k pseudonyms and a cloaking region. Consequently, identity privacy is protected by replacing the identities of requestors with pseudonyms, and location privacy is protected by replacing accurate locations of query requestors with cloaking regions. Furthermore, as an anonymity dataset includes at least k pseudonyms and a cloaking region, the association between pseudonyms and the cloaking region can be prevented at a certain degree. Likewise, the association between pseudonyms and the content of the request can also be avoided, as any pseudonym within the anonymity dataset may have issued the query request.

Spatial-temporal k-anonymity and its optimized versions are widely used in LBS snapshot queries and continuous queries [2]. To better understand the follow-up analysis of anonymity datasets, we present an example workflow of generating an anonymity dataset adopted by the modified adaptive-interval cloaking algorithm [9].

First, we present the basic definitions of an anonymity dataset for an LBS snapshot query *SnAS* = ⟨*UP*,*CR*,*TC*⟩, where *UP* = ⟨*U*_1_,*U*_2_,…,*U*_*k*_⟩ represents a set of k user pseudonyms, *CR* = ⟨*Cell*_*1*_,*Cell*_*2*_,…,*Cell*_*m*_⟩ represents a cloaking region that includes m grid cells enclosing the locations of the k users, and *TC* = 〈*TI*_1_,*TI*_2_,…,*TI*_*n*_〉 represents temporal cloaking with n time intervals of equal duration. Moreover, the time intervals 〈*TI*_1_,*TI*_2_,…,*TI*_*n*_〉 provide very little temporal information, that is, *SnAS* is a temporally-ordered sequence without a specified time.

**Fig 1:**
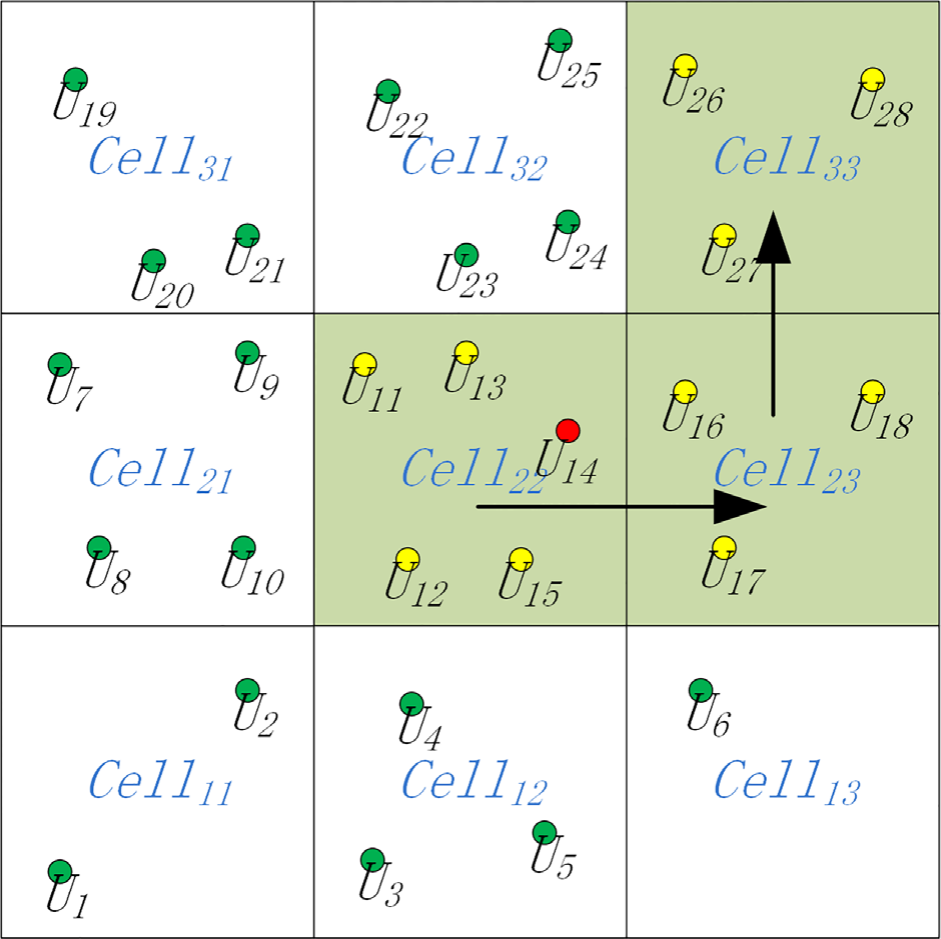
Example of an anonymity dataset for an LBS snapshot query.

Fig 1 presents an example of an anonymity dataset for an LBS snapshot query, where *SnAS* = ⟨⟨*U*_11_,*U*_12_,*U*_13_,*U*_14_,*U*_15_,*U*_16_,*U*_17_,*U*_18_,*U*_19_,*U*_26_,*U*_27_,*U*_28_⟩,⟨*Cell*_22_,*Cell*_23_,*Cell*_33_⟩,⟨1⟩⟩. We set k = 10, and for the sake of simplicity, we set the number of temporal cloaking to be 1.

**Fig 2:**
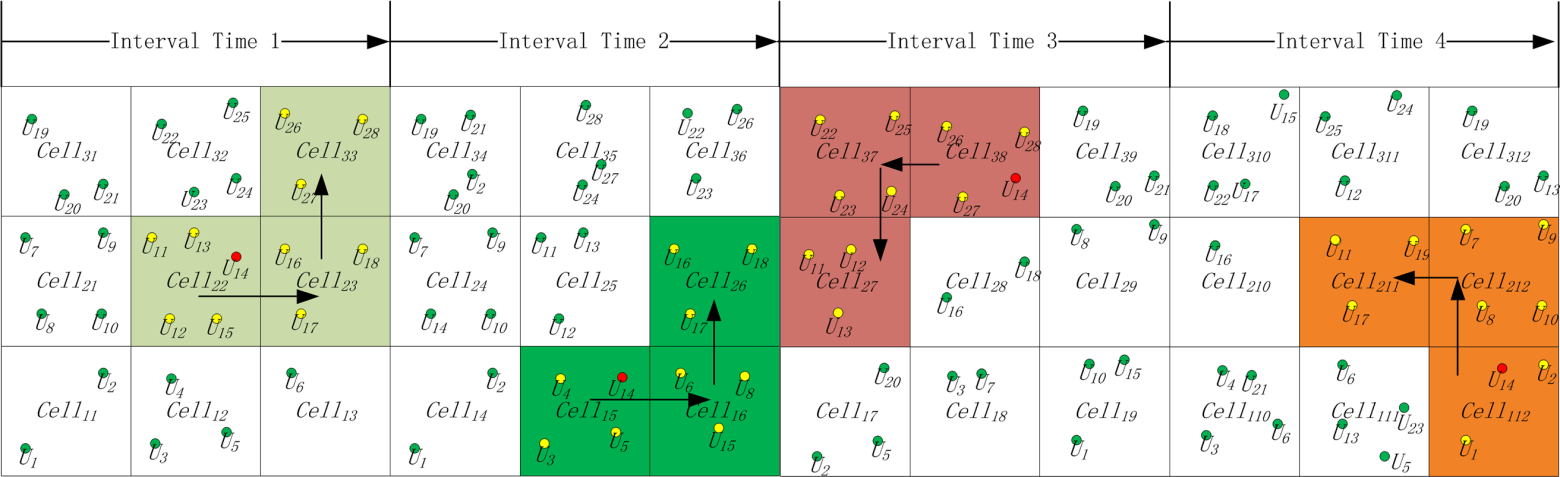
Example of an anonymity dataset for an LBS continuous query.

Based on the definitions of anonymity datasets for a snapshot query, we define an anonymity dataset for an LBS continuous query as, *CoAS* = 〈*SnAS*_1_,*SnAS*_2_,…,*SnAS*_*s*_〉 where *SnAS*_*i*_(1 ≤ *i* ≤ *s*) represents an anonymity dataset for a snapshot query. In this paper, we focus on anonymity datasets for LBS continuous queries. Fig 2 presents an example of an anonymity dataset for an LBS continuous query, where *CoAS* = 〈*SnAS*_1_,*SnAS*_2_,*SnAS*_3_,*SnAS*_4_〉,

$$\begin{array}{l} SnAS_{1}=\langle \langle U_{11},U_{12},U_{13},U_{14},U_{15},U_{16},U_{17},U_{18},U_{19},U_{26},U_{27},U_{28}\rangle ,\langle Cell_{22},Cell_{23},Cell_{33}\rangle ,\langle 1\rangle \rangle ,\\ SnAS_{2}=\langle \langle U_{3},U_{4},U_{5},U_{6},U_{8},U_{14},U_{15},U_{16},U_{17},U_{18}\rangle ,\langle Cell_{15},Cell_{16},Cell_{26}\rangle ,\langle 2\rangle \rangle ,\\ SnAS_{3}=\langle \langle U_{11},U_{12},U_{13},U_{14},U_{22},U_{23},U_{24},U_{25},U_{26},U_{27},U_{28}\rangle ,\langle Cell_{27},Cell_{37},Cell_{38}\rangle ,\langle 3\rangle \rangle ,\\ SnAS_{4}=\langle \langle U_{1},U_{2},U_{7},U_{8},U_{9},U_{10},U_{11},U_{14},U_{17},U_{19}\rangle ,\langle Cell_{112},Cell_{211},Cell_{212}\rangle ,\langle 4\rangle \rangle . \end{array}$$

Finally, as we deal only with spatial-temporal properties of anonymity datasets, an anonymity dataset for an LBS continuous query can be denoted more briefly as

$$\begin{array}{l} CoAS^{'}=\langle SnAS_{1}{}^{'},SnAS_{2}{}^{'},SnAS_{3}{}^{'},SnAS_{4}{}^{'}\rangle ,\;SnAS_{1}{}^{'}=\langle \langle Cell_{1},Cell_{2},\ldots ,Cell_{m^{1}}\rangle ,\langle TI_{1},TI_{2},\ldots ,TI_{n^{1}}\rangle \rangle ,\\ SnAS_{2}{}^{'}=\langle \langle Cell_{1},Cell_{2},\ldots ,Cell_{m^{2}}\rangle ,\langle TI_{1},TI_{2},\ldots ,TI_{n^{2}}\rangle \rangle ,\\ SnAS_{s}{}^{'}=\langle \langle Cell_{1},Cell_{2},\ldots ,Cell_{m^{s}}\rangle ,\langle TI_{1},TI_{2},\ldots ,TI_{n^{s}}\rangle \rangle . \end{array}$$

In the case of the anonymity dataset in Fig 2, the simplified notation is as follows:

$$\begin{array}{l} CoAS^{'}=\langle SnAS_{1}{}^{'},SnAS_{2}{}^{'},SnAS_{3}{}^{'},SnAS_{4}{}^{'}\rangle ,\;SnAS_{1}{}^{'}=\langle \langle Cell_{22},Cell_{23},Cell_{33}\rangle ,\langle 1\rangle \rangle ,\\ SnAS_{2}{}^{'}=\langle \langle Cell_{15},Cell_{16},Cell_{26}\rangle ,\langle 2\rangle \rangle ,\;SnAS_{3}{}^{'}=\langle \langle Cell_{27},Cell_{37},Cell_{38}\rangle ,\langle 3\rangle \rangle ,\\ SnAS_{4}{}^{'}=\langle \langle Cell_{112},Cell_{211},Cell_{212}\rangle ,\langle 4\rangle \rangle . \end{array}$$

### Location prediction method

In this section, two location prediction methods are proposed. Either follows 5 phases:

1. Mining sequential rules from anonymity datasets for LBS continuous queries;
2. Constructing transition probability matrices from the mined sequential rules;
3. Normalizing the transition probabilities in the transition probability matrices;
4. Computing n-step transition probability matrices by raising the normalized transition probability matrices to the power n;
5. Designing a rough location prediction method and an accurate location prediction method based on the n-step transition probability matrices.

### Mining sequential rules from anonymity datasets for LBS continuous queries

Prediction is an important type of data mining technology, and discovering temporal relationships in sequences of discrete events stored in large databases can help with the prediction of events[15]. Sequential patterns in sequences of events can reflect temporal relationships even without a specified time between events, and mining sequential patterns has become a popular technique for prediction. Meanwhile, as a sequential pattern only indicates that a sequence of events appears frequently in a database, it is not sufficient for the prediction of events. Thus, the concept of a sequential rule, also called a prediction rule, was proposed in [16].

A sequential rule has the form *X* → *Y*, where *X* and *Y* are two sets of events. *X* → *Y* is interpreted to mean “if events *X* appear, the events *Y* are likely to occur afterward with a given confidence value or probability”. Events *X* and events *Y* occur in succession frequently within a single sequence. A sequential rule typically has two measures of significance: support and confidence. The support of a sequential rule is here defined as the number of sequences where the left part occurs before the right part, divided by the number of sequences; the confidence of a rule is the number of sequences where the left part occurs before the right part, divided by the number of sequences where the left part occurs. For example, for a sequential rule *X* → *Y*, the support value and the confidence value of the sequential rule are respectively formulated as follows: *seq*sup(*X* → *Y*) = *seq*sup(*X* ∪ *Y*)/|*D*| and *seqconf*(*X* → *Y*) = *seq*sup(*X* ∪ *Y*) / *seq*sup(*X*), where |*D*| is the number of sequences in a sequence database *D*, *seq*sup(*X*) is the number of sequences in *D* where *X* occurs, and *seq*sup(*X* ∪ *Y*) is the number of sequences in *D* where *Y* occurs after *X*. Neither *seq*sup(*X* → *Y*) nor *seqconf*(*X* → *Y*) are less than the user-defined thresholds *seq*sup_min_ and *seqconf*_min_.

In this paper, we focus on spatial-temporal properties of anonymity datasets. Sequential rules mined from large-scale historical anonymity datasets generated by LBS continuous queries can be used to make location prediction for LBS users. In particular, a sequential rule of the form *A* → *B* with the confidence *seqsconf*(*A*– > *B*) may indicate that, if an LBS user issued a continuous query and presented an anonymous request in grid cell *A*, then with the confidence *seqsconf*(*A* → *B*) (s)he will continue to present an anonymous request in grid cell *B*. That is, sequential rules mined from anonymity datasets can reflect the movement regularity of LBS users among a series of grid cells. Table 1 presents a sample of sequential rules mined from anonymity datasets generated by LBS continuous queries.

**Table 1:** Sample of sequential rules mined from anonymity datasets generated by LBS continuous queries.

No.	Rules	*seqconf*
**1**	A->B	0.2
**2**	A->C	0.5
**3**	A->D	0.2
**4**	B->C	0.7
**5**	B->E	0.3
**6**	C->D	0.1
**7**	C->F	0.6
**8**	D->F	0.9
**9**	E->F	0.8

### Constructing n-step transition matrices by normalizing the confidence values of sequential rules

From a statistical standpoint, a mobility model for an LBS requester can be viewed as a stationary stochastic process [17]. Each movement of LBS users among a series of grid cells can be regarded as a discrete Markov process {*X*_*n*_,*n*∈*T*}, where *T* is a discrete time set (e.g., *T* = {1,2,…}), the random variable *X* represents the location of an LBS user who requests an anonymous continuous query, and *X*_*n*_ represents the value of random variable *X* at time *n* (here, *X*_*n*_ represents the grid cell where the LBS user is located at time *n*). We refer to *X*_*n*_ as a state and call *I* = {*i*_1_,*i*_2_,*i*_3_…*i*_*m*_}, the set of all possible states of *X*_*n*_, the state space of *X*_*n*_. *I* can be achieved by counting of the number of distinguished grid cells appearing in the left and right parts of the sequential rules mined from large-scale historical anonymity datasets generated by LBS continuous queries. In the case of the collection of sequential rules in Table 1, *I* = {*A*,*B*,*C*,*D*,*E*,*F*}.

For any given *n* ∈ *T*, *i*_0_,*i*_1_…*i*_*n*+1_ ∈ *I*, the discrete Markov process {*X*_*n*_,*n*∈*T*} is called a 1-order Markov chain if the following formula holds: *P*{*X*_*n*+1_ = *i*_*n*+1_ | *X*_1_ = *i*_1_,*X*_2_ = *i*_2_,…,*X*_*n*_ = *i*_*n*_} = *P*{*X*_*n*+1_ == *i*_*n*+1_ | *X*_*n*_ = *i*_*n*_}, where *P*{*X*_*n*+1_ = *i*_*n*+1_ | *X*_1_ = *i*_1_,*X*_2_ = *i*_2_,…,*X*_*n*_ = *i*_*n*_} is the conditional probability of *X*_*n*+1_ = *i*_*n*+1_ given *X*_0_ = *i*_0_, *X*_1_ = *i*_1_,…,*X*_*n*_ = *i*_*n*_, and *P*{*X*_*n*+1_ == *i*_*n*+1_ | *X*_*n*_ = *i*_*n*_} is the conditional probability of *X*_*n*+1_ = *i*_*n*+1_ given *X*_*n*_ = *i*_*n*_. That is, a 1-order Markov chain {*X*_*n*_,*n*∈*T*} can be characterized as memoryless: the next state *X*_*n*+1_ = *i*_*n*+1_ depends only on the current state *X*_*n*+1_ = *i*_*n*+1_ but not on the sequence of events that preceded it. In the case of sequential rules mined from anonymity datasets, the memoryless means that the future grid cell at which an LBS user arrives is independent of all but the most recent grid cell.

The conditional probability *P*{*X*_*n*+1_ == *i* | *X*_*n*_ = *j*}, *i*, *j* ∈ *I* can also be taken as a one-step transition probability from state *i* to state *j*, which is denoted by *p*_*ij*_. In statistical significance, *p*_*ij*_ is consistent with the confidence value of the sequence rule of the form *i* → *j*. Based on all one-step transition probabilities that corresponding to the sequential rules, a transition matrix $P=\left\{\begin{array}{cccc} p_{11} & p_{12} & \ldots & p_{1m}\\ p_{21} & p_{22} & \ldots & p_{2m}\\ \ldots & \ldots & \ldots & \ldots \\ p_{m1} & p_{m2} & \ldots & p_{mm} \end{array}\right\}$ can be constructed, where the dimension *m* is equal to the number of states in the state space *I*. Continuing with the sequential rules in Table 1, the generated transition matrix is: $P=\left[\begin{array}{ccccccc} & A & B & C & D & E & F\\ A & 0 & 0.2 & 0.5 & 0.2 & 0 & 0\\ B & 0 & 0 & 0.7 & 0 & 0.3 & 0\\ C & 0 & 0 & 0 & 0.1 & 0 & 0.6\\ D & 0 & 0 & 0 & 0 & 0 & 0.9\\ E & 0 & 0 & 0 & 0 & 0 & 0.8\\ F & 0 & 0 & 0 & 0 & 0 & 0 \end{array}\right]$.

However, the transition matrix must be normalized so that the condition $\sum_{j\in I}p_{ij}=1,i\in I$ holds. For example, for given *i* = *A*, *i* ∈ {*A*,*B*,*C*,*D*,*E*,*F*}, $\sum_{A}p_{Aj}$ must be equal to 1, while $\sum_{A}p_{Aj}$ = *p*_*AA*_ + *p*_*AB*_ + *p*_*AC*_ + *p*_*AD*_ + *p*_*AE*_ + *p*_*AF*_ = 0 + 0.2 + 0.5 + 0.2 + 0 + 0 = 0.9.

The normalization formula of *p*_*ij*_ is $p_{ij}{}^{‘}=p_{ij}/\sum_{j\in I}p_{ij}$, $p_{A\text{i}}{}^{‘}=p_{Ai}/\sum_{i=1}^{5}p_{Ai}$. Then, we get $p_{AA}^{'}=0$, $p_{AB}^{'}=0.2222$, $p_{AC}^{'}=0.5556$, $p_{AD}^{'}=0.2222$, $p_{AE}^{'}=0$ and $p_{AF}^{'}=0$. We refer to a transition matrix with normalized transition probabilities as a one-step transition matrix, and denote by *P*^(1)^ the result corresponding to *P*^0^.

In addition, the normalized probabilities are time-invariant. One reason for this is that the confidence values of the sequential rules mined from large-scale historical anonymity datasets generated by LBS continuous queries essentially reflect routine behaviors of a large number of LBS users. On the other hand, sequential rules only reflect temporally-ordered relationships between routine behaviors without specifying times. That is, LBS users follow a common route regardless of when they move. Hence, the Markov chain {*X*_*n*_,*n*∈*T*}, which corresponds to the movement of LBS users among a series of grid cells, can also be characterized as time-invariant, and further *P*^(1)^ can be considered to be independent of *n*. Furthermore, we can obtain the n-step transition matrix *P*^(*n*)^ from *P*^(1)^ using the formula *P*^(*n*)^ = (*P*^(1)^)^*n*^. The maximum value of *n* can be determined from the conditions that *P*^(*n*)^ is not a zero matrix and that *n* is less than the length of the longest sequence of LBS anonymity datasets. Here, by raising *P*^(1)^ to an appropriate power, we obtain *P*^(2)^, *P*^(3)^, and *P*^(4)^ as follows:

$$\begin{array}{l} P^{\left(1\right)}=\left[\begin{array}{ccccccc} & A & B & C & D & E & F\\ A & 0 & 0.2222 & 0.556 & 0.2222 & 0 & 0\\ B & 0 & 0 & 0.7 & 0 & 0.3 & 0\\ C & 0 & 0 & 0 & 0.1429 & 0 & 0.8571\\ D & 0 & 0 & 0 & 0 & 0 & 1\\ E & 0 & 0 & 0 & 0 & 0 & 1\\ F & 0 & 0 & 0 & 0 & 0 & 0 \end{array}\right],\;P^{\left(2\right)}=\left[\begin{array}{ccccccc} & A & B & C & D & E & F\\ A & 0 & 0 & 0.1555 & 0.0794 & 0.0667 & 0.6984\\ B & 0 & 0 & 0 & 0.1000 & 0 & 0.9000\\ C & 0 & 0 & 0 & 0 & 0 & 0.1429\\ D & 0 & 0 & 0 & 0 & 0 & 0\\ E & 0 & 0 & 0 & 0 & 0 & 0\\ F & 0 & 0 & 0 & 0 & 0 & 0 \end{array}\right],\\ P^{\left(3\right)}=\left[\begin{array}{ccccccc} & A & B & C & D & E & F\\ A & 0 & 0 & 0 & 0.0222 & 0 & 0.2794\\ B & 0 & 0 & 0 & 0 & 0 & 0.1000\\ C & 0 & 0 & 0 & 0 & 0 & 0\\ D & 0 & 0 & 0 & 0 & 0 & 0\\ E & 0 & 0 & 0 & 0 & 0 & 0\\ F & 0 & 0 & 0 & 0 & 0 & 0 \end{array}\right],\;P^{\left(4\right)}=[\begin{array}{ccccccc} & A & B & C & D & E & F\\ A & 0 & 0 & 0 & 0. & 0 & 0.0222\\ B & 0 & 0 & 0 & 0 & 0 & 0\\ C & 0 & 0 & 0 & 0 & 0 & 0\\ D & 0 & 0 & 0 & 0 & 0 & 0\\ E & 0 & 0 & 0 & 0 & 0 & 0\\ F & 0 & 0 & 0 & 0 & 0 & 0 \end{array}]. \end{array}$$

### Prediction for arriving at a target location based on n-step transition matrices

#### Rough prediction

This prediction consists of three main phases:

First, we specify a grid cell as the target location. Continuing the examples of sequential rules in Table 1, we assume that the grid cell represented by F is the target location.

Second, from the n-step transition matrix, we directly derive paths along which LBS users can arrive at the target location with specified probabilities. As the paths only include the target location and the grid cell ("begin location" for short) where the LBS users are when they begin to move, we denote these paths as simple paths. Based on the transition matrices *P*^(1)^ ∼ *P*^(4)^, we obtain all simple paths by ascending number of steps as shown in Table 2.

**Table 2:** Simple paths for arriving at a target location *F*.

Steps	Begin location	Probability	Target location
**1**	D	1	F
	C	0.8571	
	E	1	
**2**	A	0.6984	
	B	0.9000	
	C	0.1429	
**3**	A	0.2749	
	B	0.10000	
**4**	A	0.0222	

Finally, by matching the grid cell ("current location" for short) where an LBS user is currently with all simple paths, we can make a location prediction for LBS users’ arriving at the target location. In the case of the simple paths in Table 2, the location prediction results are shown in Table 3. For example, when an LBS user appears in grid cell *A*, three predictions (indicated by the shaded entries) can be performed. In particular, after leaving the grid cell *A*, the LBS user has the three probability values 0.6984, 0.2749 and 0.0222 for arriving at the target location *F* through 2-, 3-, and 4-step transitions respectively. Likewise, location prediction can be performed when LBS users occupy grid cells *B*, *C*, *D* and *E*.

**Table 3:** Rough prediction for arriving at a target location *F*.

Current location	Steps	Probability	Target location
A	2	0.6984	F
	3	0.2749	
	4	0.0222	
B	2	0.9000	
	3	0.10000	
C	1	0.8571	
	2	0.1429	
D	1	1	
E	1	1	

As can be seen in Table 3, the transitions from the current location to the target location can be classified as either single step or multistep. For single step transitions, the paths that LBS users follow are shown clearly. In particular, after leaving their current location, an LBS user arrives directly at the target location. For example, after leaving grid cell *C*, an LBS user arrives directly at the target location *F* with probability 0.8571.

However, for multistep transitions, we find that the simple paths that LBS users follow include one or more intermediate locations, but these intermediate locations are unknown, so the detailed path between the current location and the target location cannot be investigated. For example, LBS users currently in grid cell *C* arrive at the target location *F* with probability 0.1429 through a 2-step transition. This simple path certainly includes one intermediate location, but we cannot know the intermediate location. If there are several options for the intermediate location, then the simple path actually contains several detailed paths, and the probability 0.1429 is the sum of the probabilities for those detailed paths. In many practical applications, it is significant to know these detailed paths to predict future movements of the LBS users [18].

#### Accurate prediction

We propose a method of calculating probabilities for detailed paths to make accurate location predictions. The principle of calculating transition probabilities for detailed paths is to iteratively calculate the probabilities for detailed paths with (*S*+1) steps by combining the probabilities for detailed paths with *n*−1 steps and the probabilities of simple paths with *n* steps.

The pseudo code for calculating transition probabilities for detailed paths is given below.

**Algorithm** 1: *R*: *CalcuDetailPath*(*L*,*TL*)

**Input:** *L*, a linked list of 1- to n-step transition matrices; *TL*, a target location.

**Output:** *R*, a linked list of detailed paths with one to n transition steps.

1.   **{**   *P*^(1)^ = *L*.*Get*(1);

2.        $R_{simp\_in}^{\left(1\right)}=P^{\left(1\right)}.GetArrive(TL)$;

3.        $R_{Detail\_in}^{\left(1\right)}=R_{simp\_in}^{\left(1\right)}$;

4.        $R.add\left(R_{Detail\_in}^{\left(1\right)}\right)$;

5.        $CalcuDetailPathI\left(L,TL,R_{Detail\_in}^{\left(1\right)},1,R\right)$;

6.        Return *R*;

7.    }

**Algorithm** 2: *CalcuDetailPathI*(*L*,*TL*,*R*_*Exac_in*_,*S*,*refR*)

**Input:** *L*, a linked list of 1-step to n-step transition matrices; *TL*, a target location; *R*_*Detail_in*_, a linked list of detailed paths with *S*-step transition steps; *S*, the transition steps in the current iteration; *R*, a parameter passed by reference, which represents a linked list of detailed paths with one to *S* transition steps.

**Output:** null.

1.   {   *P*^(*S*+1)^ = *L*.*Get*(*S* + 1);

2.        $R_{simp\_in}^{\left(S+1\right)}=P^{\left(S+1\right)}.GetArrive(TL)$;

3.        For $\left(i=1;i<=R_{simp\_in}^{\left(S+1\right)}\cdot count;i++\right)$

4.      {  $\left(E_{S+1}=R_{simp\_in}^{\left(S+1\right)}\cdot Get(i)\cdot firstState\right)$;

5.            For (*j* = 1; *j* ≤ *R*_*Datai_in*_⋅*count*;*j*++)

6.            {    *prob*_1_ = *R*_*Detai_in*_⋅*Get*(*j*)⋅*probValue*;

7.            *E*_*S*_ = *R*_*Detai_in*_⋅*Get*(*j*)⋅*FirstState*;

8.              If (*P*^(1)^⋅*Exist*(*E*_*S*+1_,*E*_*S*_))

9.                  { Pr*ob*_2_ = *P*^(1)^⋅Pr*obValue*(*E*_*S*+1_,*E*_*S*_);

10.                  $R_{Detai\_in}^{\left(S+1\right)}\cdot addprob(prob_{1}\times prob_{2})$;

11.                  $Subs=R_{Detai\_in}^{\left(S+1\right)}\cdot Get(j)\cdot Subs$;

12.                  $R_{Detai\_in}^{\left(S+1\right)}\cdot addPath(E_{S+1},Subs)$;

13.            }//end if

14.        }//end for

15.    }//end for

16.    $R_{Detai\_in}=R_{Eetai\_in}^{\left(S+1\right)}$;

17.    *R*⋅*add*(*R*_*Detai_in*_);

18.    *S*++;

19.    If (*S* ≤ *L*⋅*count*)

20.    *CalcuDetaiPathI*(*L*,*TL*,*R*_*Detai_in*_,*S*,*R*);

21. }

**Algorithm 1** is the main procedure. Lines 1~4 are the initialization, where the simple paths with one transition step are obtained from *P*^(1)^; line 5 calls the sub-procedure **Algorithm 2** to obtain a linked list of detailed paths with one to n transition steps; line 6 returns the final result *R*.

**Algorithm 2** performs recursive operations. Line 1 takes an (*S*+1)-step transition matrix *P*^(*S*+1)^ from a linked list of transition matrices *L*; line 2 obtains the simple paths $R_{simp\_in}^{\left(S+1\right)}$ with one transition step from *P*^(*S*+1)^; lines 3~15 combine $R_{simp\_in}^{\left(S+1\right)}$ with the passed parameter *R*_*Detai_in*_ to obtain the detailed paths with (*S*+1) steps; line 8 checks for the state pair (*E*_*S*+1_,*E*_*S*_), and line 10 calculates the probabilities for all detailed paths in $R_{{}_{Detai\_in}}^{\left(S+1\right)}$; lines 16~17 assign $R_{{}_{Detai\_in}}^{\left(S+1\right)}$ to *R*_*Detai_in*_; lines 18~19 check whether (*S*+2) is greater than the number of steps in the linked list *L*; line 20 passes *R*_*Detai_in*_ for the next recursive call of procedure *CalcuDetailPathI*.

**Fig 3:**
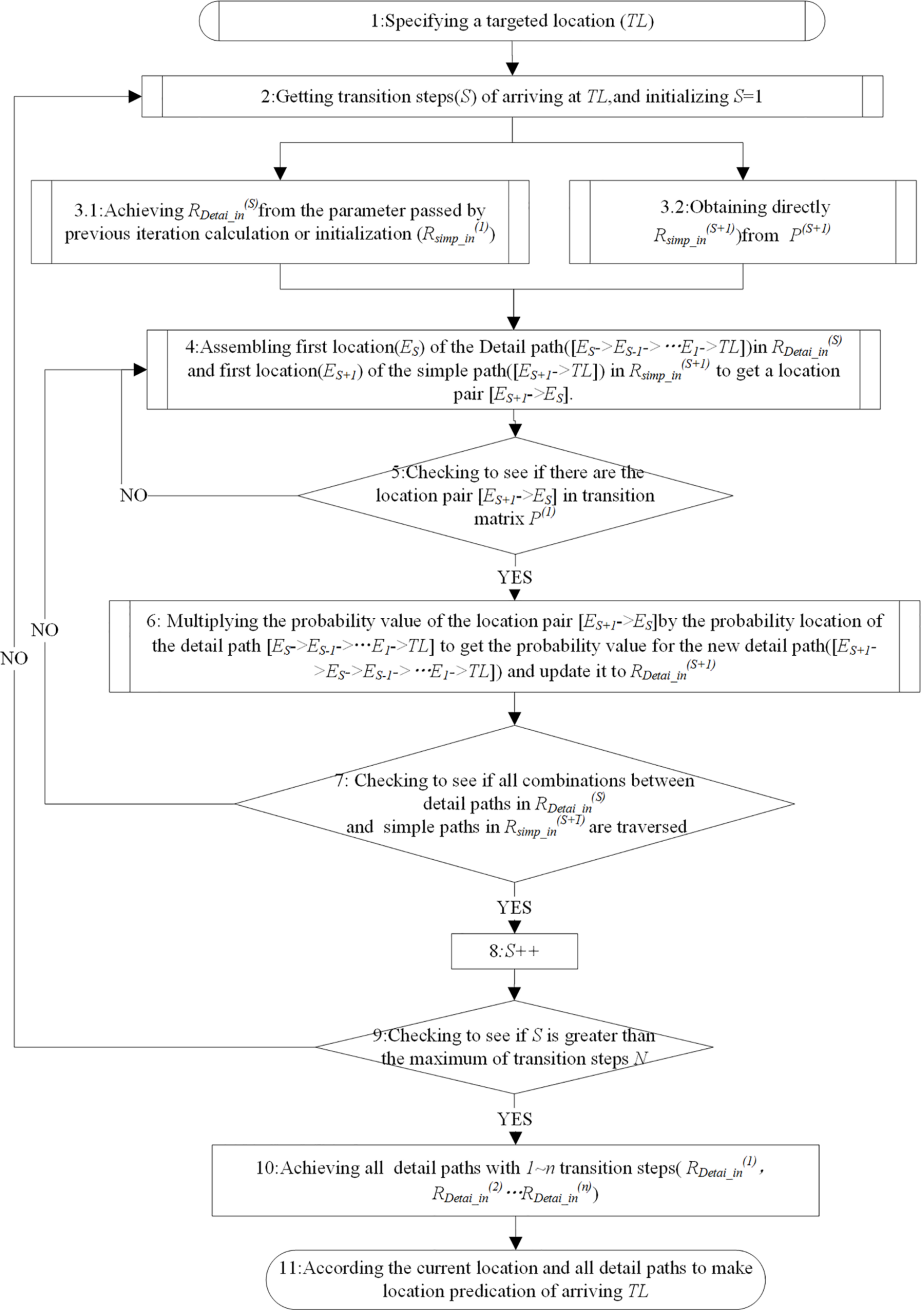
Flowchart for making accurate location predictions based on the probabilities of detailed paths.

Next, we present the flowchart for the two algorithms and accurate location prediction based on the detailed paths obtained. The flowchart is depicted in Fig 3. The processes of the workflow are described below.

(1) As in the rough prediction, we first specify a grid cell as the target location. We again assume that grid cell *F* is the target location.

(2) From the (*S*+1)-step transition matrix, we directly obtain all simple paths and the probabilities of arriving at *F* after (*S*+1) steps. Here, we obtain all simple paths with two steps from *P*^(2)^ and record them in $R_{simp\_in}^{\left(2\right)}$, so that $R_{simp\_in}^{\left(2\right)}$ = ([*A* → *F*], [*B* → *F*], [*C* → *F*]).

(3) Detailed paths with *S* steps are determined from the parameter passed by the previous iteration or by the initialization. Here, we initialize to obtain detailed paths with one step $R_{Detai\_in}^{\left(1\right)}$ from simple paths with one step $R_{simp\_in}^{\left(1\right)}$ obtained from *P*^(1)^, so that $R_{Exac\_in}^{\left(1\right)}$ = $R_{simp\_in}^{\left(1\right)}$ = ([*C* → *F*], [*D* → *F*], [*E* → *F*]).

(4) Assemble the start locations of the simple paths and the start locations of the detailed paths to obtain location pairs. For each start location of the detailed paths in $R_{Detai\_in}^{\left(1\right)}$ and each start location of the simple paths in $R_{simp\_in}^{\left(2\right)}$, we can obtain a location pair. For example, for the detailed path ([*C* → *F*] and the simple path [*A* → *F*], we obtain the location pair [*A* → *C*]. Likewise, we can obtain other location pairs: [*B* → *C*], [*C* → *E*]. Furthermore, we obtain ([*A* → *D*], [*B* → *D*], [*C* → *D*], [*A* → *E*], [*B* → *E*] and [*C* → *E*].

(5) Check to see whether there are location pairs in *P*^(1)^ that place the start locations of the simple paths at the head of the detailed paths to obtain new detailed paths with n transition steps. Here, we obtain the location pairs [*A* → *C*], [*A* → *D*], [*B* → *C*] and [*C* → *D*], and the new detailed paths [*A* → *C* → *F*], [*A* → *C* → *F*], [*B* → *C* → *F*] and [*C* → *D* → *F*].

(6) Multiply the probabilities of the location pairs by the probabilities of the detailed paths with *S* steps to obtain probabilities for the detailed paths with (*S*+1) steps. Here, By multiplying the probability 0.8571 for the detailed path [*C* → *F*] and the probability 0.5556 for the location pair [*A* → *C*], we obtain the probability 0.4762 for the detailed path [*A* → *C* → *F*]. Similarly, we obtain probabilities 0.2222, 0.59997, 0.3, and 0.1429 for the detailed paths [*A* → *D* → *F*], [*B* → *C* → *F*], [*B* → *E* → *F*] and [*C* → *D* → *F*], respectively.

(7) Iterate to find detailed paths and probabilities for (*S* + 2) transition steps until the maximum number of transition steps is reached. Here, we first obtain all simple paths $R_{simp\_in}^{\left(3\right)}$ = ([*A* → *F*], [*B* → *F*]) from *P*^(3)^, then combine these with $R_{Detai\_in}^{\left(2\right)}$ = ([*A* → *C* → *F*], [*A* → *D* → *F*], [*B* → *C* → *F*], [*B* → *E* → *F*], [*C* → *D* → *F*]) in steps (2)~(6) and obtain $R_{Exac\_in}^{\left(3\right)}$ = ([*A* → *B* → *C* → *F*], [*A* → *B* → *E* → *F*], [*A* → *C* → *D* → *F*], [*B* → *C* → *D* → *F*]).

Furthermore, we can combine $R_{Detai\_in}^{\left(3\right)}$ with $R_{simp\_in}^{\left(4\right)}=[A\to F]$ to obtain $R_{Detai\_in}^{\left(4\right)}$ = [*A* → *B* → *C* → *D* → *E*]. Finally, we obtain all detailed paths and probabilities of arriving at *F*, as shown in Table 4.

**Table 4:** Detailed paths for arriving at the target location *F*.

Steps	Current location	Detailed path	Probability	Target location
**1**	D	D->F	1	F
	C	C->F	0.8571	
	E	E->F	1	
**2**	A	A->C->F	0.4762	
		A->D->F	0.2222	
	B	B->C->F	0.59997	
		B->E->F	0.3	
	C	C->D->F	0.1429	
**3**	A	A->B->C->F	0.1333	
		A->B->E->F	0.0666	
		A->C->D->F	0.0794	
	B	B->C->D->F	0.10003	
**4**	A	A->B->C->D->F	0.0222	

(8) From the detailed paths and probabilities, we can make accurate location predictions for arriving at the target location. The accurate location prediction results are shown in Table 5. After leaving grid cell B, an LBS user will arrive at the target location F with probability 0.59997 along the detailed *B*, *C*, *D* [*B* → *C* → *F*], with probability 0.3 along the detailed path [*B* → *E* → *F*], and probability 0.10003 along the detailed path [*B* → *C* → *D* → *F*] (indicated by the shaded entries). Likewise, accurate location prediction can be performed when LBS users start from grid cells *A*, *C*, *D* and *E*.

**Table 5:** Accurate prediction for arriving at the target location F.

Current location	Steps	Detailed path	Probability	Target location
A	2	A->C->F	0.4762	F
		A->D->F	0.2222	
	3	A->B->C->F	0.1333	
		A->B->E->F	0.0666	
		A->C->D->F	0.0794	
	4	A->B->C->D->F	0.0222	
B	2	B->C->F	0.59997	
		B->E->F	0.3	
	3	B->C->D->F	0.10003	
C	1	C->F	0.8571	
	2	C->D->F	0.1429	
D	1	D->F	1	
E	1	E->F	1	

## Experiments and Discussion

### Data preparation

#### Simulated anonymity datasets for LBS continuous queries

Because spatial-temporal k-anonymity and its variants have not been widely applied in business LBS systems, we adopt a software system developed in the literature to simulate large-scale anonymity datasets for LBS continuous queries from GPS trajectories. Table 6 summarizes the basic characteristics of the simulated datasets.

**Table 6:** Basic characteristics of simulated anonymity datasets for LBS continuous queries.

Parameter	Value
Number of sequences	490
Maximum number of cloaking regions in a single sequence	38
Minimum number of cloaking regions in a single sequence	2
Avg. number of cloaking regions for all sequences	19.8
Minimum number of time intervals(h) in a single sequence	1
Maximum number of time intervals (h) in a single sequence	12
Range of each cell of cloaking regions (m^2^)	105*88
Number of cells contained by all cloaking regions	692

#### Sequential rules & n-step transition matrix

We adopt the RuleGrowth algorithm in SPMF [19] to mine sequential rules from simulated anonymity datasets. The parameters *seq*sup_min_ and *seqconf*_min_ are set to be 0.02 and 0.24, respectively. The 18 mined sequential rules are given in Table 7.

**Table 7:** Sequential rules mined from simulated anonymity datasets for LBS continuous queries.

No.	Sequential rule	Confidence value
**1**	D->M	0.3023
**2**	A->E	0.2619
**3**	B->E	0.2703
**4**	C->E	0.2500
**5**	D->E	0.2791
**6**	F->E	0.2424
**7**	D->N	0.2558
**8**	H->E	0.3125
**9**	E->I	0.3182
**10**	F->I	0.2424
**11**	G->I	0.2608
**12**	O->N	0.2537
**13**	H->I	0.3125
**14**	J->I	0.2500
**15**	P->N	0.2821
**16**	K->N	0.2500
**17**	K->I	0.3250
**18**	L->I	0.2439

By normalizing the confidence values of the 18 mined sequential rules, we obtain the one-step transition matrix *P*_*simu*_^(1)^, and further calculate the 2-step transition matrix *P*_*simu*_^(2)^.

$$P_{simu}^{\left(1\right)}=\left(\begin{array}{ccccccccccccccccc} & A & B & C & D & E & F & G & H & I & J & K & L & M & N & O & P\\ A & 0 & 0 & 0 & 0 & 1 & 0 & 0 & 0 & 0 & 0 & 0 & 0 & 0 & 0 & 0 & 0\\ B & 0 & 0 & 0 & 0 & 1 & 0 & 0 & 0 & 0 & 0 & 0 & 0 & 0 & 0 & 0 & 0\\ C & 0 & 0 & 0 & 0 & 1 & 0 & 0 & 0 & 0 & 0 & 0 & 0 & 0 & 0 & 0 & 0\\ D & 0 & 0 & 0 & 0 & 0.3333 & 0 & 0 & 0 & 0 & 0 & 0 & 0 & 0.3611 & 0.3056 & 0 & 0\\ E & 0 & 0 & 0 & 0 & 0 & 0 & 0 & 0 & 1 & 0 & 0 & 0 & 0 & 0 & 0 & 0\\ F & 0 & 0 & 0 & 0 & 0.5 & 0 & 0 & 0 & 0.5 & 0 & 0 & 0 & 0 & 0 & 0 & 0\\ G & 0 & 0 & 0 & 0 & 0 & 0 & 0 & 0 & 1 & 0 & 0 & 0 & 0 & 0 & 0 & 0\\ H & 0 & 0 & 0 & 0 & 0.5 & 0 & 0 & 0 & 0.5 & 0 & 0 & 0 & 0 & 0 & 0 & 0\\ I & 0 & 0 & 0 & 0 & 0 & 0 & 0 & 0 & 0 & 0 & 0 & 0 & 0 & 0 & 0 & 0\\ J & 0 & 0 & 0 & 0 & 0 & 0 & 0 & 0 & 1 & 0 & 0 & 0 & 0 & 0 & 0 & 0\\ K & 0 & 0 & 0 & 0 & 0 & 0 & 0 & 0 & 0.5652 & 0 & 0 & 0 & 0 & 0.4348 & 0 & 0\\ L & 0 & 0 & 0 & 0 & 0 & 0 & 0 & 0 & 1 & 0 & 0 & 0 & 0 & 0 & 0 & 0\\ M & 0 & 0 & 0 & 0 & 0 & 0 & 0 & 0 & 0 & 0 & 0 & 0 & 0 & 0 & 0 & 0\\ N & 0 & 0 & 0 & 0 & 0 & 0 & 0 & 0 & 0 & 0 & 0 & 0 & 0 & 0 & 0 & 0\\ O & 0 & 0 & 0 & 0 & 0 & 0 & 0 & 0 & 0 & 0 & 0 & 0 & 0 & 1 & 0 & 0\\ P & 0 & 0 & 0 & 0 & 0 & 0 & 0 & 0 & 0 & 0 & 0 & 0 & 0 & 1 & 0 & 0 \end{array}\right)$$

$$P_{simu}^{\left(2\right)}=\left(\begin{array}{ccccccccccccccccc} & A & B & C & D & E & F & G & H & I & J & K & L & M & N & O & P\\ A & 0 & 0 & 0 & 0 & 0 & 0 & 0 & 0 & 1 & 0 & 0 & 0 & 0 & 0 & 0 & 0\\ B & 0 & 0 & 0 & 0 & 0 & 0 & 0 & 0 & 1 & 0 & 0 & 0 & 0 & 0 & 0 & 0\\ C & 0 & 0 & 0 & 0 & 0 & 0 & 0 & 0 & 1 & 0 & 0 & 0 & 0 & 0 & 0 & 0\\ D & 0 & 0 & 0 & 0 & 0 & 0 & 0 & 0 & 0.3333 & 0 & 0 & 0 & 0 & 0 & 0 & 0\\ E & 0 & 0 & 0 & 0 & 0 & 0 & 0 & 0 & 0 & 0 & 0 & 0 & 0 & 0 & 0 & 0\\ F & 0 & 0 & 0 & 0 & 0 & 0 & 0 & 0 & 0.5 & 0 & 0 & 0 & 0 & 0 & 0 & 0\\ G & 0 & 0 & 0 & 0 & 0 & 0 & 0 & 0 & 0 & 0 & 0 & 0 & 0 & 0 & 0 & 0\\ H & 0 & 0 & 0 & 0 & 0 & 0 & 0 & 0 & 0.5 & 0 & 0 & 0 & 0 & 0 & 0 & 0\\ I & 0 & 0 & 0 & 0 & 0 & 0 & 0 & 0 & 0 & 0 & 0 & 0 & 0 & 0 & 0 & 0\\ J & 0 & 0 & 0 & 0 & 0 & 0 & 0 & 0 & 0 & 0 & 0 & 0 & 0 & 0 & 0 & 0\\ K & 0 & 0 & 0 & 0 & 0 & 0 & 0 & 0 & 0 & 0 & 0 & 0 & 0 & 0 & 0 & 0\\ L & 0 & 0 & 0 & 0 & 0 & 0 & 0 & 0 & 1 & 0 & 0 & 0 & 0 & 0 & 0 & 0\\ M & 0 & 0 & 0 & 0 & 0 & 0 & 0 & 0 & 0 & 0 & 0 & 0 & 0 & 0 & 0 & 0\\ N & 0 & 0 & 0 & 0 & 0 & 0 & 0 & 0 & 0 & 0 & 0 & 0 & 0 & 0 & 0 & 0\\ O & 0 & 0 & 0 & 0 & 0 & 0 & 0 & 0 & 0 & 0 & 0 & 0 & 0 & 0 & 0 & 0\\ P & 0 & 0 & 0 & 0 & 0 & 0 & 0 & 0 & 0 & 0 & 0 & 0 & 0 & 0 & 0 & 0 \end{array}\right)$$

### Results and Discussion

#### Experiment 1

**Fig 4:**
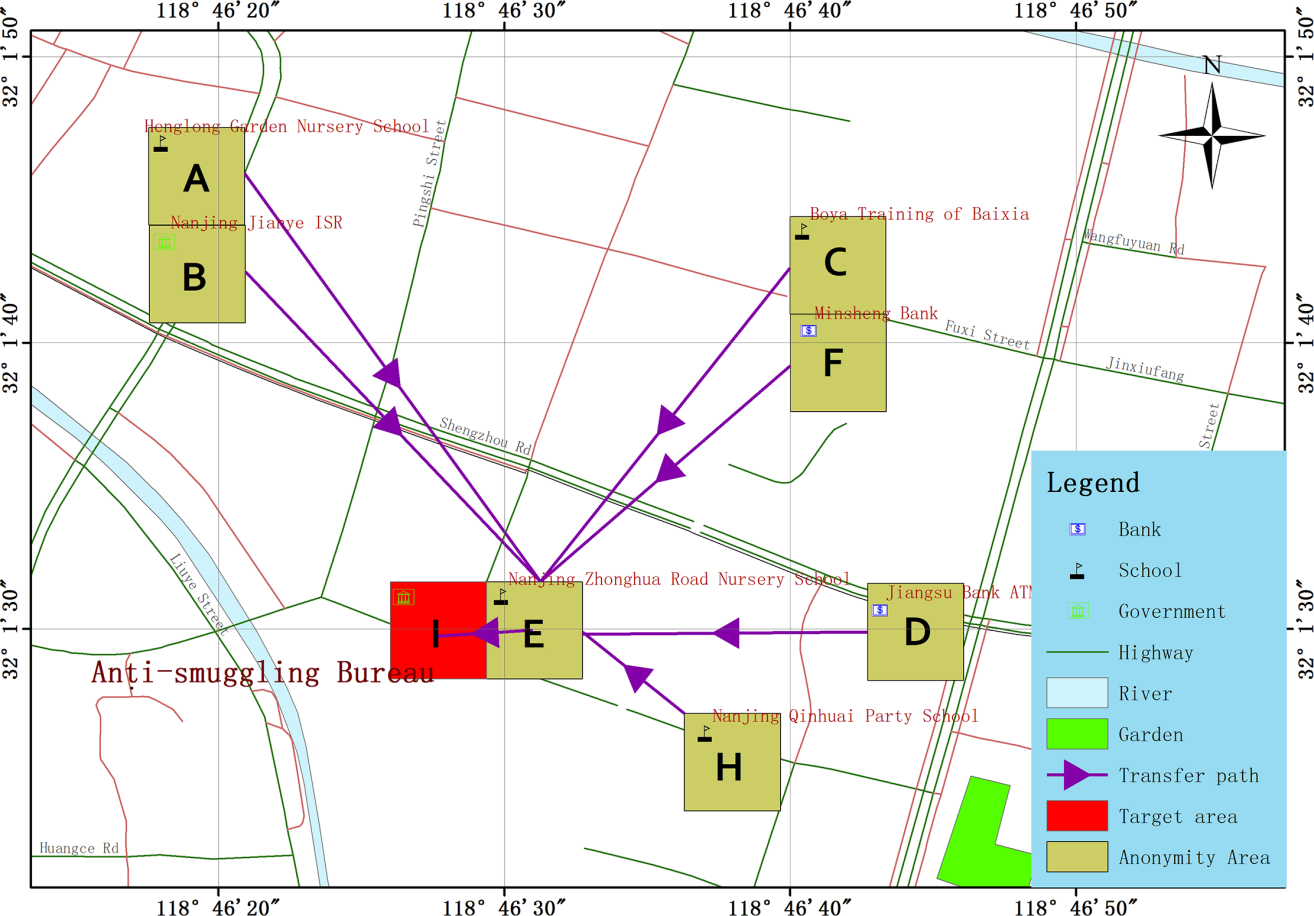
Mapping display of accurate location predictions on geographic background datasets.

We specify the grid cell *I* as the target location, and derive simple paths for arriving at *I* from *P*_*simu*_^(1)^ and *P*_*simu*_^(2)^ directly. Based on the simple paths, we make the rough location predictions shown in Table 8. Furthermore, we obtain detailed paths using the algorithm *CalcuDetailPath*, and make accurate location predictions based on the detailed paths. The results are shown in Table 9 and are mapped onto geographic background datasets in Fig 4.

**Table 8:** Rough predictions for arriving at the target location *I*.

Current location	Steps	Probability	Target location
A	2	1	I
B	2	1	
C	2	1	
D	2	0.3333	
E	1	1	
F	1	0.5	
	2	0.5	
G	1	1	
H	1	0.5	
	2	0.5	
J	1	1	
K	1	0.5652	
L	1	1	

**Table 9:** Accurate predictions for arriving at the target location *I*.

Current location	Steps	Detailed path	Probability	Target location
A	2	A->E->I	1	**I**
B	2	B->E->I	1	
C	2	C->E->I	1	
D	2	D->E->I	0.3333	
F	2	F->E->I	0.5	
H	2	H->E->I	0.5	

#### Experiment 2

**Fig 5:**
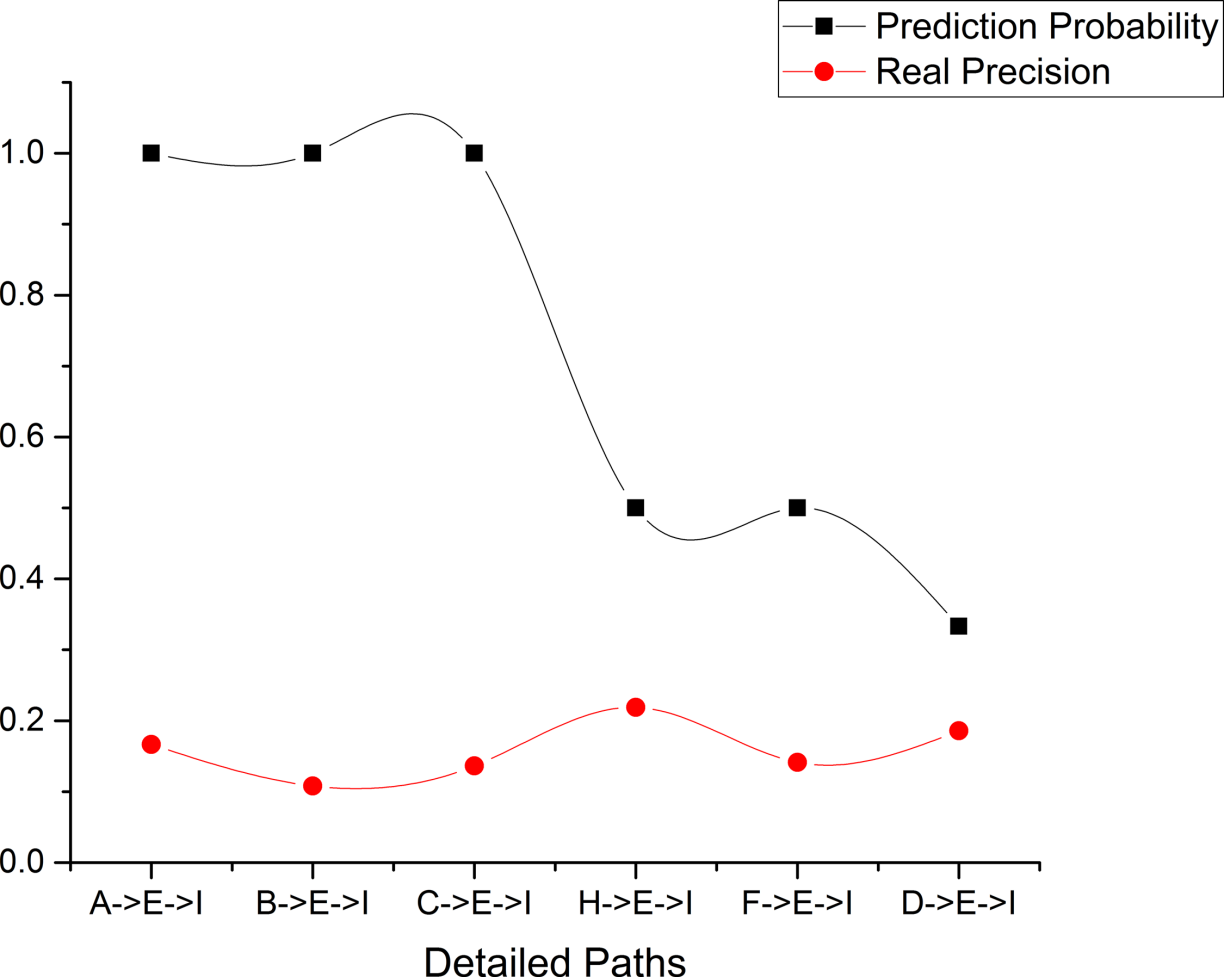
Comparison between real precision and location prediction probabilities.

This experiment aims to verify the correctness of the proposed location prediction methods. As mentioned above, we find that the accurate prediction method is essentially an optimized version of the rough location prediction method. Thus, we only evaluate the correctness of the accurate prediction method with the *realprecision* measure. This measure is a direct measurement calculated as the number of correct predictions divided by the total number of predictions. For example, for the detailed path [*A* → *E* → *I*], the *realprecision* value is equal to the conditional probability *P*(*A* → *E* → *I*|*A*). The results of this experiment are shown in Fig 5, from which we see that for the detailed paths [*H* → *E* → *I*], [*F* → *E* → *I*] and [*D* → *E* → *I*], the *realprecision* measure and the prediction probability are similar, while for the detailed paths [*A* → *E* → *I*], [*B* → *E* → *I*] and [*C* → *E* → *I*], there are significant differences between the *realprecision* values and the prediction probabilities. Namely, the *realprecision* values are much lower than the prediction probabilities.

Next, we analyze causes of this problem. According to the algorithm *CalcuDetailPath*, we find that the prediction probabilities for the detailed paths [*A* → *E* → *I*], [*B* → *E* → *I*] and [*C* → *E* → *I*] are products of the probabilities for the detailed path [*E* → *I*] and the location pairs [*A* → *E*], [*B* → *E*] and [*C* → *E*] in *P*_*simu*_^(1)^.

Furthermore, we find from Table 7 and *P*_*simu*_^(1)^ that there are significant differences between the confidence values and the normalized values for the sequential rules [*A* → *E*], [*B* → *E*], [*C* → *E*] and [*E* → *I*]. Specifically, the confidence values are 0.2619, 0.2703, 0.2500, and 0.3182, respectively, but the normalized values are all 1. We argue that reason is that the confidence threshold *seqconf*_min_ for sequential rule mining is too large to allow the discovery of enough sequential rules. Hence, we make the assumption that too large confidence threshold for sequential rules may result in significant differences between the *realprecision* values and the prediction probabilities for the detailed paths [*A* → *E* → *I*], [*B* → *E* → *I*], [*C* → *E* → *I*]. Next, we further test this hypothesis with **Experiment 3**.

#### Experiment 3

First, we use the two lower confidence thresholds 0.2 and 0.22 to mine sequential rules and obtain 104 sequential rules and 56 sequential rules respectively, among which the sequential rules with start locations A, B, C, and E are shown in Table 10.

**Table 10:** Sequential rules with confidence thresholds 0.2 and 0.22 and start locations A, B, C, and E.

*seqconf*_min_	Begin	Sequential rule	Confidence value
**0.2**	**A**	A->E	0.2619
		A->I	0.2381
		A->N	0.2381
		A->M	0.2381
	**B**	B->E	0.2703
	**C**	C->E	0.2500
		C->I	0.2273
	**E**	E->I	0.2353
		E->N	0.2059
		E->M	0.2059
**0.22**	**A**	A->E	0.2619
		A->I	0.2381
		A->N	0.2381
		A->M	0.2381
	**B**	B->E	0.2703
	**C**	C->E	0.2500
		C->I	0.2273
	**E**	E->I	0.2353

**Fig 6:**
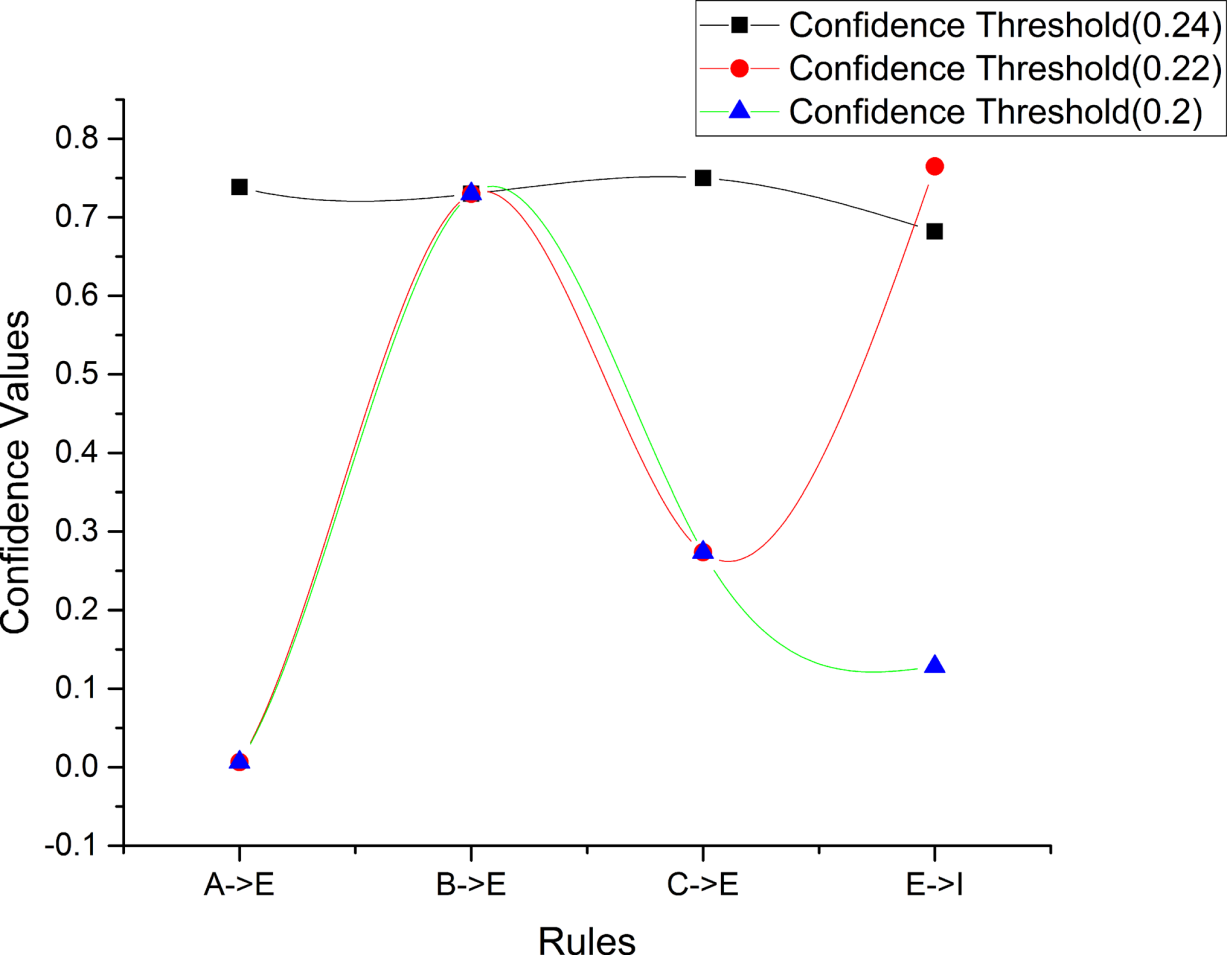
Comparison of differences between confidence values and normalized values for the sequential rules with confidence thresholds 0.2, 0.22 and 0.24.

Next, we obtain normalized values for the confidence values of sequential rules (in Table 10) of [*A* → *E*], [*B* → *E*], [*C* → *E*] and [*E* → *I*], which are shown in Table 11, and the comparison of the differences between the confidence values and the normalized values is shown in Fig 6. We see that differences decrease along with decreasing confidence thresholds except in the case of the sequential rule [*B* → *E*].

**Table 11:** Sequential rules with confidence thresholds 0.2 and 0.22 and normalized confidence values.

*seqconf*_min_	Sequential rule	Confidence value	Normalized value
0.2	A->E	0.2619	0.2683
	B->E	0.2703	1
	C->E	0.25	0.5238
	E->I	0.2353	0.3636
0.22	A->E	0.2619	0.2683
	B->E	0.2703	1
	C->E	0.25	0.5238
	E->I	0.2353	1

**Fig 7:**
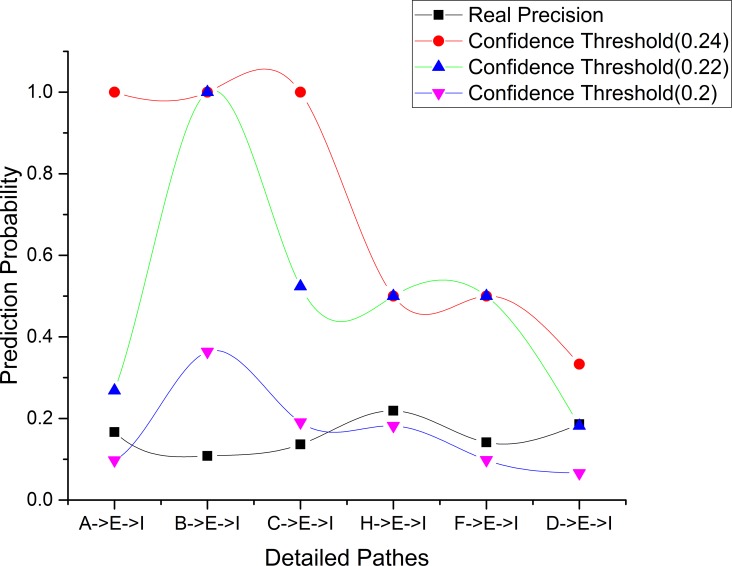
Comparison of varying proximities between location prediction probabilities and *realprecision* values for the sequential rules with confidence thresholds 0.2, 0.22 and 0.24.

Finally, by constructing n-step transition matrices and adapting the algorithm *CalcuDetailPath*, we obtain location prediction probabilities for the detailed paths [*A* → *E* → *I*], [*B* → *E* → *I*], [*C* → *E* → *I*]. The comparison of varying proximities between location prediction probabilities and *realprecision* values is shown in Fig 7.

We see that as the confidence threshold decreases, the location prediction probabilities for six of the detailed paths are all closer to their corresponding *realprecision* values.

This experiment confirms our previous hypothesis from **Experiment 2**. Thus, we can conclude that proximity between location prediction probabilities and *realprecision* values for detailed paths can be adjusted flexibly by setting different confidence thresholds for mining sequential rules. That is, when users believe that the accuracy of the accurate prediction cannot meet their requirements, they can obtain higher prediction accuracy by decreasing confidence thresholds of mining sequence rules used to construct transition probability matrices.

## Conclusion and future work

Because of its ease of implementation, spatial-temporal k-anonymity has become a mainstream approach for protecting the privacy of LBS users. Analyzing large-scale anonymity datasets can benefit some LBS applications. In this paper, we propose two location prediction methods for the probabilities of arriving at specified locations based on transition probability matrices constructing from sequential rules for spatial-temporal k-anonymity dataset. By conducting extensive experiments, we have verified the correctness and flexibility of our proposed methods.

However, because technologies are intent neutral, they harbor neither benevolent nor malevolent intent with respect to the individuals using them. Thus, our proposed location prediction methods can also lead to substantial privacy threats. For example, target locations that are regarded as privacy-sensitive regions, such as military zones, red-light districts, and so on, may be susceptible to more menacing attacks, because the existing spatial-temporal k-anonymity methods and its variants mainly concern the current and historical private information of LBS users but not the future information [20]. Hence, in the future, we will study the capabilities and limitations of those attacks methods to lay foundations for research into performance optimization for spatial-temporal k-anonymity methods and its variants, thus helping data miners and domain experts ensure that privacy-sensitive knowledge is released or accessible only to trusted parties.

## Supporting Information

S1 Text Seqsup0.02_seqconf0.2. Sequential rules mined with parameters *seq*sup_min_ and *seqconf*_min_ set to be 0.02 and 0.20, which are used in Experiment 3.
(TXT)

S2 Text Seqsup0.02_seqconf0.2_1step. Accurate one-step location predictions for arriving at the target location *I*, which are used in Experiment 3.
(TXT)

S3 Text Seqsup0.02_seqconf0.2_2step. Accurate two-step location predictions for arriving at the target location *I*, which are used in Experiment 3.
(TXT)

S4 Text Seqsup0.02_seqconf0.2_3step. Accurate three-step location predictions for arriving at the target location *I*, which are used in Experiment 3.
(TXT)

S5 Text Seqsup0.02_seqconf0.22. Sequential rules mined with parameters *seq*sup_min_ and *seqconf*_min_ set to be 0.02 and 0.22, which are used in Experiment 3.
(TXT)

S6 Text Seqsup0.02_seqconf0.22_1step. Accurate one-step location predictions for arriving at the target location *I*, which are used in Experiment 3.
(TXT)

S7 Text Seqsup0.02_seqconf0.22_2step. Accurate two-step location predictions for arriving at the target location *I*, which are used in Experiment 3.
(TXT)

S8 Text Seqsup0.02_seqconf0.22_3step. Accurate three-step location predictions for arriving at the target location *I*, which are used in Experiment 3.
(TXT)

S9 Text Seqsup0.02_seqconf0.24. Sequential rules mined with parameters *seq*sup_min_ and *seqconf*_min_ set to be 0.02 and 0.24, which are used in Experiment 1.
(TXT)

S10 Text Seqsup0.02_seqconf0.24_1step. Accurate one-step location predictions for arriving at the target location *I*, which are used in Experiment 1 and 2.
(TXT)

S11 Text Seqsup0.02_seqconf0.24_2step. Accurate two-step location predictions for arriving at the target location *I*, which are used in Experiment 1 and 2.
(TXT)

S12 Text Test datasets. Test datasets are to evaluate the correctness of the accurate prediction method with the *realprecision* measure, which are used in Experiment 2 and 3.
(TXT)
